# Translation and initial cross-cultural adaptation of the tool for support-gradual return-to-work for persons with chronic musculoskeletal pain to the Swedish setting

**DOI:** 10.3233/WOR-230665

**Published:** 2024-10-07

**Authors:** Gunilla M. Liedberg, Mathilda Björk, Marie-France Coutu, Marie-José Durand, Christina Turesson

**Affiliations:** a Department of Health, Medicine and Caring Sciences, Division of Prevention, Rehabilitation and Community Medicine, Linköping University, Linköping, Sweden; b Department of Health, Pain and Rehabilitation Centre, Medicine and Caring Sciences, Linköping University, Linköping, Sweden; c School of Rehabilitation, Faculty of Medicine and Health Sciences, Université de Sherbrooke, Longueuil, Quebec, Canada

**Keywords:** Return-to-work, vocational rehabilitation, chronic pain, instrument development, cross cultural comparison, occupational therapist

## Abstract

**BACKGROUND:**

A well-defined and clear procedure is a key factor supporting return-to-work and enhancing collaboration and understanding between employers and employees. The adaptation of the Tool for Support-Gradual Return to Work, TS-GRTW, addresses relevant cultural aspects valuable for wider adoption.

**OBJECTIVE:**

develop a Swedish version, the GRTWswe, for implementation and integration into the Swedish labor market’s RTW process. This involved translating, culturally adapting, and assessing the appropriateness and utility.

**METHODS:**

In the initial step, a double back translation was performed to create an initial translated version. This version was then utilized in individual consultations, accompanied by an agreement questionnaire. For the subsequent step, group consultations were held to refine and customize the tool to suit the Swedish context. Ten occupational therapists completed the questionnaires, with mean agreement scores surpassing three on a four-point scale. Out of these, nine participated in group consultations.

**RESULTS:**

The findings suggest the requirement for specific modifications to the GRTWswe. These adaptations are essential because of cultural differences in organizational structures and reference frameworks. Moreover, participants unanimously agreed to broaden the scope of target groups, encompassing employees without regard for diagnosis and expanding the range of professions that can utilize this tool. This step aims to enhance the tool’s applicability and usefulness.

**CONCLUSIONS:**

The study found strong alignment between questionnaire responses and group consultations outcomes, affirming the adapted tool’s suitability for use in a Swedish context. The tool benefits employers and employees by enhancing communication, encouraging collaboration, and structuring processes, promising lasting improvements to work conditions.

## 1 Introduction

Chronic musculoskeletal pain (CMSP) lasting over 3 months, including neck/shoulder and back pain or generalized widespread pain such as fibromyalgia (FM), affects 10.4% [1] to 20% of adults [2–4]. CMSP hinders daily activities, including employment and workdays lost [3]. Work-related issues result in negative outcomes for employees, reduced productivity for both employees and employers, and has a significant impact on society [5]. Individuals with chronic pain commonly face disruptions in their work tasks, which may manifest in cognitive challenges, like difficulty focusing, affective issues such as reduced tolerance, or physical limitations like decreased stamina [6].

Reintegrating individuals suffering from chronic pain into work is challenging, demanding collaborative efforts from various stakeholders. In Sweden, this includes officers from the Swedish Social Insurance Agency (SSIA) and Swedish Public Employment Service (SPES) who may be engaged in vocational rehabilitation. Additionally, employers, case managers from Occupational Health Services, rehabilitation coordinators, occupational therapists (OTs), and healthcare physicians are part of the Return-To-Work (RTW) process in healthcare, as referenced in this study [7]. The SSIA assess the need for RTW interventions and coordinates the process with other stakeholders [7]. Research on stakeholders’ experiences found that health professionals’ efforts to facilitate individuals’ RTW were hindered by a rigid system, excessive bureaucracy, and a lack of coordination and collaboration among involved parties. This highlights the importance of each stakeholder in understanding their roles and responsibilities in the RTW process [8].

In 2019, Sweden introduced broader employer responsibility guidelines aimed at helping employees with illnesses to remain active in the labor market. This expanded employer responsibility entails facilitating the process by adjusting job tasks and the work environment, along with the obligation to document a rehabilitation plan [7]. Support from the employer, for example, in the form of increased cooperation through regular contact between the workplace and the employee was reported as a facilitator of RTW, while absence of support was considered a barrier [9].

Furthermore, on a broader international scale, employers from various jurisdictions have indicated that they lack the necessary tools to effectively manage the RTW process [10]. Individuals with CMSP often face insufficient support from employers during RTW [11], and this sentiment is also reported by employers themselves [12, 13] Workplace factors, like adjustments and strategies, positive colleagues, and employers, outweigh individual predictors for RTW [9]. Timely problem reporting and open communication between employers and employees are crucial. Frequently, essential accommodations can be pinpointed through discussions between the employer and the employee [14]. Communication gaps about pain may stem from employees’ assumptions regarding consequences or employers’ willingness to talk about it. For instance, employees may fear the possible consequences of sharing their problems with management. The extent to which employers are willing to discuss pain is linked to the overall openness within the organizational culture regarding the topic. Employers’ competences in pain management are also likely to play a significant role. Some employers express a lack of confidence in their abilities to support employees with pain. Employers also express a desire for more structured conversations with employees about the work environment and pain. This suggests that establishing formal structures for discussing pain issues could be advantageous for pain prevention [12]. Using a structured tool such as GRTWswe can be a solution to fulfilling the employer’s responsibility [7] required by the employer in Sweden.

There is a need for Swedish-specific instruments for the RTW process that foster collaboration and structured work situation reviews, which are easy to follow up. The adaptation of a Canadian tool to address relevant cultural aspects is useful for wider adoption. A well-defined and clear procedure for RTW is a key factor supporting RTW [15], as it enhances collaboration and understanding between employers and employees [16]. The Tool for Support-Gradual Return to Work (TS-GRTW) can aid in this RTW process [10].

The concept Margin of Manoeuvre (MM) emphasizes the importance of allowing employees to develop effective work methods that meet production requirements without compromising their health. This concept underpins the designed tool [15, 17]. The TS-GRTW includes a 21 pages instruction guide and a two-sided planning worksheet. Ten sections build up the planning worksheet and the headings are as follows: 1, restrictions/recommendations issued by the attending physician; 2, planned work schedule; 3, work tasks identified; 4, productivity expected; 5, anticipated obstacles or difficulties; 6, ways to offset these difficulties; 7, worker’s level of confidence regarding the work plan for the week; and 8, signatures. These eight sections are completed at the beginning of the week. The remaining sections, 9–10, are answered at the end of the week and are entitled: 9, attainment of production objectives; and 10, increase in discomfort level [10].

The instruction guide consists of a description of the origins of the tool and its underlying concepts. Furthermore, a brief description in the form of answers to the questions “for whom”, “when”, “how”, and “why”, as well as its limitations are included. Finally, a figure illustrating each step of the TS-GRTW is followed by examples, instructions, and practical advice [10].

Supervisors’ ability to support RTW is closely linked to communication, policies, and organizational factors, with their attitudes playing a key role in the process [18]. The planning worksheet is designed for joint use by employees and employers, promoting regular follow-up and enhancing collaboration. It helps counteract passivity on both sides during a RTW process.

Our objective was to develop a Swedish version—the GRTWswe—for implementation and integration into the Swedish labor market’s RTW process. This involved translating, culturally adapting, and assessing the appropriateness and utility of a Canadian tool called TS-GRTW with occupational therapists (OTs) active in RTW in areas with individuals with CMSP.

The concepts of appropriateness and utility refer to the perceived fit, relevance, or compatibility of the innovation or evidence-based practice for a given practice setting, provider, or consumer; and/or perceived fit of the innovation to address a particular issue or problem [19], and the clinical relevance, support for decision making, ability to facilitate/improve communication, application, potential for implementation, and transferability [20].

## 2 Methods

### 2.1 Ethical considerations

This study adhered to the Declaration of Helsinki [21]. Participants received detailed information about the study, with the assurance of voluntary participation and the option to withdraw. Written informed consent was obtained, and data were kept confidential in a secure database. The study was approved by the Swedish Ethical Review Board (Dnr 2021-06678-01).

### 2.2 Study design

A preliminary step used a double back translation of the guide to obtain a preliminary translated version. This version was used for the next step—an individual consultation—using an agreement questionnaire. For the last step, group consultations were conducted with the same participants to collect information and to clarify and adapt the tool to a Swedish context.

To respect the intellectual property rights of the tool, first, the approval of the original authors was obtained for translation into Swedish on 2021-02-19.

### 2.3 Translation and cross-cultural adaptation

Translation and adaptation of TS-GRTW (instruction guide and planning worksheet) to Swedish conditions followed the steps described by Beaton et al. [22]. The steps are 1) translation, 2) synthesis, 3) translation back into the original language, 4) review by expert committee (consultations in this study), and 5) pre-testing (step 5 is not performed in this study but in a study currently underway).

Two project authors (GML, CT) translated and culturally adapted the tool, taking structural differences in the Swedish RTW process and societal involvement into consideration. We modified the text for clarity and mutual understanding, with both authors synthesizing the translations. Furthermore, a patient representative with chronic pain, who had minimal work disability, reviewed the synthesized version to ensure to ensure everyday language use, following the recommended naïve translation approach by Beaton et al. [22]. The patient representative also assessed the instruction guide and planning sheet for potential ambiguities and item understandability. Afterwards, a backward translation was performed by professional translators, and after a review of two of the Swedish authors of the back-translated text, it was sent to the developers of the TS-GRTW (MFC; MJD) for clarification and conditions of acceptability.

### 2.4 Participants

According to Beaton’s translation method [22], a review/consultation was conducted by an expert committee with ten OTs with a minimum of one year of experience assisting individuals with CMSP in RTW or enhancing their current work conditions.

Snowball sampling [23] was used to reach OTs working with persons with chronic pain and their RTW process. One OT employed at the chronic pain specialist unit at a university hospital, one OT in primary care, and one OT in occupational health care (OHC) were asked for participation in the study. They served as the initial contacts for study participants, with OTs recommending other OTs who met the study criteria, and these, in turn, could suggest potential participants. The OTs constituted an expert committee [23], where views were collected on the first version of the Swedish version of TS-GRTW (GRTWswe). In all, thirteen OTs were asked to participate in the study.

### 2.5 Procedure

#### 2.5.1 Individual consultation

Participants received the preliminary Swedish version of GRTWswe by mail along with a project information letter, a consent form, and an agreement rating questionnaire. The questionnaire also included demographic questions about their work area, years working with chronic pain clients, and the participant’s age. The questionnaire was the same one that was used in the study where TS-GRTW was designed and tested [10]. In the agreement rating questionnaire, which was completed individually, the participant estimated how well the statement of criteria regarding the tool matched their own opinion on a 4-point scale. That is, they were asked to evaluate each of the items in terms of comprehensibility, wording, interpretations, cultural issues, and clarification. The grading ranged from ‘do not disagree at all (1)’ to ‘fully agree (4)’, applied to a total of 21 statements, and the tool was assessed based on four assessment areas in terms of its appropriateness and utility: the content of this section is pertinent, essential and sufficient; the content of this section is clear and well formulated; the content of this section is clear and pertinent; and the visual appearance of this section makes it easy to use.

In cases where grading the correspondence between the participant’s perception and the tool was stated to be equivalent to ‘2’ or lower, participants were asked to suggest improvements for whatever was considered problematic. Furthermore, any suggestions for improvement could be made in the comments box. The questionnaire was returned in the provided envelope. They were also told to retain a copy of their questionnaire before submission, in order to have it available during the upcoming group consultation.

After the questionnaires had been received, a descriptive quantitative analysis was completed of sociodemographic data (age, gender, in what area they work with persons with chronic pain, how long the participant worked with people with chronic pain) and the compliance figures for each claim from the agreement questionnaire.

#### 2.5.2 Group consultation

Two weeks after the last questionnaire submission, a Zoom group consultation was scheduled with participants to discuss proposed changes to the cross-culturally adapted GRTWswe tool. Due to scheduling challenges, two separate group sessions were offered instead of a single gathering.

For the 21 statements on which participant had to rate their level of agreement on comprehensibility, wording, interpretations, and cultural issues on a group level, no parts of the tool received a disagreement score (grading equal to or lower than 3). Aside from overall group agreement, there were issues that the authors felt needed to be clarified and deepened based on the completed agreement questionnaires: the concept of a rehabilitation plan; the planning sheet; the biopsychosocial perspective; time perspective on follow-up; clinical relevance; and finally, space was also left in the discussions to encourage participants to raise other concerns.

A week before the group meeting, participants received a semi-structured interview document via email, which included their individual proposals, changes, and reflections based on the comments from the agreement questionnaire. Both groups received the same set of template questions.

The first author moderated the groups, while the last author served as a co-moderator responsible for taking written notes. These Zoom interviews were recorded, with each group consultation lasting 75 and 48 minutes, respectively. Both were conducted in June 2022.

### 2.6 Analysis

The first author (GML) manually coded the data. A qualitative content analysis using a directed approach [24] was conducted on written comments from the agreement questionnaire, written memos from group consultations, and recordings. The analysis focused on identifying themes related to content, format, usability, comprehension, and language within the predetermined structure of appropriateness and utility. The analysis was reviewed by the last author (CT) to ensure it reflected the three data sources and by comparing, reviewing, discussing, and refining, a consensus between the two authors was achieved. During the analysis process, all relevant domains were assigned quotes, from the Agreement questionnaires (written) and from the recorded group consultations, to allow one to evaluate the results. Quotations were transformed into written language according to Kvale [25] to prevent stigmatization. In the Results section, brackets [] are used to indicate implied words.

## 3 Results

Thirteen OTs initially agreed to participate, but three later withdrew due to work-related time constraints. Ten participants completed the questionnaire, and among these, one chose not to take part in the group consultation. Consequently, nine OTs attended the group sessions, with five in the first meeting and four in the second.

Four OTs worked in specialist care (Pain and Rehabilitation Clinics), four in primary care, and two in Swedish Public Employment Service (SPES). Participants’ mean age was 49 years, ranging from 42 to 57 years, and the mean length of time participants had spent collaborating with persons with chronic pain and RTW was 9 years, ranging from 1.3 to 22 years. The participants represented five geographical areas in Sweden. Characteristics of the participants are presented in Table 1.

**Table 1:** Demographical data of participants

Workspace	Geographical area	Age	Number of years	Participated in
			worked in the field	group
			of chronic pain	consultation
Specialist care in chronic pain	Healthcare region: Mid Sweden	50	1,5	Yes, group 2
Specialist care in chronic pain	Region Stockholm	43	10	Yes, group 2
Specialist care in chronic pain	Southeastern health care region	51	22	Yes, group 1
Specialist care in chronic pain	Southeastern health care region	50	1,3	Yes, group 1
Swedish Public Employment Service	Östergötland	47	10	Yes, group 1
Swedish Public Employment Service	Skåne Unity Southwest	45	12	Yes, group 1
Primary care unit	Southeastern health care region (Central unit)	53	16	No
Primary care unit	Southeastern health care region (West unit)	52	9	Yes, group 2
Primary care unit	Southeastern health care region (East unit)	42	3	Yes, group 2
Primary care unit	Southeastern health care region (East unit)	57	7	Yes, group 1

Health care OTs have a broad range of responsibilities, including reconciliation meetings with various parties such as SSIA, physicians, and rehabilitation coordinators. They also engage in direct collaboration meetings with employers. These professionals visit workplaces to assess needs and develop strategies for individuals to RTW successfully or to establish a sustainable work situation. The OTs at SPES have reported assessing work ability within real workplace settings and conduct numerous on-site visits to evaluate and implement workplace adjustments and test different work aids.

Table 2 displays the mean agreement scores from the expert committee, the ten OTs; all of whom scored >3. However, comments were provided concerning content, wording, and contextual aspects within the Swedish setting.

**Table 2:** Results of the agreement rating questionnaire

Wording of the statements	Section concerned	Page	Mean score (SD)
The content of the section is pertinent,	Background and summary	2	3.8 (0.42)
essential and sufficient	At a glance	3–4	3.4 (0.70)
	Observe	4	3.6 (0.52)
	Step 1 (initial meeting with the worker)	6	3.7 (0.67)
	Step 2 (instructions for sections 1–8)	7–10	3.5 (0.71)
	Step 3 (executing the working plan)	11	3.8 (0.42)
	Step 4 Evaluate the working week	11–12	4.0 (0)
	Step 5 Return to ordinary work	13	3.6 (0.52)
	Planning worksheet for the GRTW	Appendix	3.5 (0.71)
The content of this section is clear and well	Background and summary	2	3.5 (0.71)
formulated	At a glance	3–4	3.5 (0.53)
	Observe	4	3.7 (0.48)
	Step 1 (initial meeting with the worker)	6	3.5 (0.53)
	Step 2 (instructions for sections 1–8)	7–10	3.7 (0.48)
	Step 3 (executing the working plan)	11	3.5 (0.53)
	Step 4 Evaluate the working week	11–12	3.7 (0.48)
	Step 5 Return to ordinary work	13	3.9 (0.32)
The content of this section is clear and	Figure illustrating the GRTW process.	5	3.9 (0.32)
pertinent	Appendix 1 (example of ways to increase the MM)	14	3.6 (0.52)
The visual appearance of this section makes	Planning worksheet for the GRTW	Appendix	3.5 (0.53)
it easy to use	Full manual	1–14	3.8 (0.42)

The group consultations revealed a new category—Format and Clarity—which addressed language, wording, tool length, and visual appeal in addition to the existing categories of Appropriateness and Utility.

### 3.1 Format and clarity

All the participants shared the view that the Instruction guide was far too comprehensive in scope, and suggestions emerged to develop a shorter guide, one comprising at most 2–3 pages, to increase the likelihood of it being read.

“Generally, way too much text. I think that above all, the overview will be used”. (Primary care, South-eastern health care region)

The overview of the process, included in the Instruction guide, was regarded as easy to understand, and as being noticeably clear and helpful for seeing the total process. Still, clarifications were needed:

“What are musculoskeletal disorders? \dots  even if you have a diagnosis, it is not sure the person [employer] understands that this [diagnosis] belongs to the category ‘musculoskeletal disorders’ \dots  ” (SPES, Skåne Unity Southwest)

The planning worksheet was generally well-received, and seen as easy to understand, complete, and structured. To enhance user-friendliness, some words should be replaced with more worker- and employer-friendly language, promoting ease of use.

“These can be hard-to-understand words for language-impaired/low-skilled people”. (SPES, Skåne Unity Southwest).

Space for more text was requested in the planning worksheet, as well as a digital version with ‘growing’ fill boxes. Furthermore, suggestions emerged regarding how a digital version of the planning worksheet could be developed in the future.

“In a digital version, it can be good to have explanation boxes/tips that come up”. (SPES, Skåne Unity Southwest)

### 3.2 Appropriateness

The example of the completed planning worksheet, which featured fictional individuals and workplaces, sparked extensive discussions within the groups. Participants felt that the worksheet primarily focused on physical challenges and stressed the need to equally consider the psychosocial perspective.

“Should it only be used for persons with pain who do not have any major problems with psychosocial factors? Often there are completely varied factors than the pain itself that constitute the major obstacle to returning to work, stress, anxiety, conflicts between employers/colleagues/employees, low motivation, or unwillingness to go back, fatigue, cognitive problems, etc.” (Primary care, southeastern healthcare region).

The planning worksheet was seen as helpful in clarifying the employees’ duties, expectations, and concerns. Participants suggested adding a line in the ‘planned work schedule’ for possible breaks, emphasizing their importance for recovery and the need for mutual agreement.

“Clearly the break will be an agreement if it is also included in the planning sheet”. Specialist care, healthcare region: Mid Sweden)

The ‘level of discomfort’ included in the Planning worksheet prompted major discussions in both groups. The existing scale with four scale steps from ‘unchanged’ to ‘greatly increased [discomfort]’ raised questions such as:

“Strange that you can’t estimate whether [your level of discomfort] decreases”. (SPES, Östergötland)

and

“Sometimes it is hard to define unchanged, slightly decreased etc \dots  what it really means”. (SPES, Skåne Unity Southwest).

Suggestions to use a scale of eleven steps from ‘no inconvenience’ to ‘worst possible discomfort’ emerged. According to the participants, this would facilitate a comparison between the separate occasions when the scale was filled in and allow the scale to also show improvement.

The term ‘rehabilitation plan’ in the instruction guide raised concerns in the groups due to varying interpretations among RTW professionals in Sweden. There was a need to rephrase and clarify the concept for better understanding.

“In the best of worlds, it would have been good if there had been a common plan regarding gradual return to work and other daily activities \dots  ” (special care, Region Stockholm).

Discussion arose in the groups regarding SSIA and their role and responsibilities in RTW. Participants expressed concerns that the concept used in the instruction guide could easily be confused with a rehabilitation plan from healthcare professionals. Suggestions appeared to change the text in the instruction guide to:

“Based on the plan of rehabilitation from the SSIA”. (special care, healthcare region: Mid Sweden).

The underlying concept of MM was discussed in the groups and regarded by a few participants as quite a novel concept.

“[It’s] a bit of a difficult word, you don’t really use it”. (SPES, Östergötland).

However, some participants found the concept valuable and believed it provided an advantageous regulatory framework, as per the Swedish Work Environment Authority.

“I like the concept ‘margin of manoeuvre but it may need further explanation as it is complex. Some [persons with chronic pain] may experience they do not have any margin of manoeuvre [at work] at all  \dots   but they may have some opportunities to decide how to plan their work, when to take breaks, or how to vary their work assignments”. (specialist care, southeastern healthcare region)

### 3.3 Utility

The GRTWswe tool was regarded as very relevant and easy to use by all participants for documenting the difficulties an employee may experience in a workplace and what needs to be addressed in a structured way.

“It’s a strength that it [the GRTWswe] is concrete, you sign it, the timeline is defined, and you talk about the steps that otherwise may be lost when the person comes back to work”. (SPES, Skåne Unity Southwest).

This tool was seen to enhance communication by promoting mutual, regular contact between actors, and fostering collaborative development through ongoing communication.

“Generally speaking, [this is] good material to support return to work. Should be easy to use for employers and employees [to use] together”. (primary care, southeastern healthcare region)

Both groups stressed the need for broader use, suggesting that making the tool available to a wider range of labor market groups beyond chronic pain conditions would increase its utility.

“Specific words such as ‘musculoskeletal conditions’ should be able to be removed”. (specialist care, Region Stockholm). Another aspect of usability was raised by OTs from SPES, indicating that the tool could be valuable for individuals in on-the-job training and in transitioning to regular employment. They suggested that sharing the responsibility for planning between the administrator and the individual, using the planning sheet, could enhance participation.

“Can the area of application [of the GRTWswe] be broader and also include vocational rehabilitation for unemployed persons such as work training?” (SPES, Skåne Unity Southwest).

In terms of applicability, participants emphasized that this tool could be used not only by employer representatives but also by professionals involved in the RTW process. They indicated, for example, occupational therapists active in rehabilitation programs at both specialist level as well as primary care level. Participants considered that this will be a plan that goes hand in hand with the documented rehabilitation plan created by healthcare professionals.

“Occupational therapists can use it as part of the transition and do it together with employers or rehabilitation coordinators or whoever is now involved in the process”. (Primary care, South-eastern healthcare region). The specialist care team, involving multiple professions, primarily releases patients with limited follow-up. Participants noted that rehabilitation coordinators would take over for more extensive follow-up. The tool could serve as an initial handover tool to the next party. Participants stressed the challenge for employers in identifying task-related difficulties and how to manage them.

“We release the patients and do not have much follow-up, but you can perhaps use it in a first planning stage with the employer where you make a plan. The planning sheet should be written together with the team where several are engaged in what should be done. Psychologists and/or physicians should be there.” (special care, Region Stockholm)

In the group discussions, a debate about who should formulate restrictions and recommendations in the planning work sheet was emphasized. Participants found it challenging to use restrictions, as they are uncommon in Sweden; instead, recommendations are typically highlighted in documents.

“It’s perhaps better to have two separate boxes \dots  to make it clear that a proposal [for instance avoid repetitive lifting] \dots  is a recommendation or a restriction”. (specialist care, southeastern healthcare region).

As for restrictions to be filled in in the planning sheet, it is also important to emphasize who should be responsible for this, and according to the participants, this should come from healthcare professionals.

“It is important to clarify who fills in the restrictions and that there is an assessment that forms the basis for them”. (primary care, southeastern healthcare region).

Concerns arose that restrictions could discourage individuals from going beyond them, even though it might be effective, and that consequences could be significant. One example of this potential outcome was mentioned:

“It’s easy to get caught up in the fact that I’m absolutely not allowed to work with my arms above my head, etc.” (primary care, southeastern healthcare region).

### 3.4 Summary of a Swedish version of GRTW for further testing

Based on the findings regarding format, clarity, appropriateness, and utility we propose the following sections and content of the Swedish version of GRTW (Table 3).

**Table 3:** The sections and content of the translated and culturally adapted version of the TS-GRTWswe planning worksheet for creating an individual workplan. Sections 1–8 are filled in at the first occasion and sections 9–10 are used to follow up on the workplan

Sections	Content
1. Recommendations and restrictions given by healthcare professionals	Text box for listing recommendations and restrictions
2. Planned work schedule	Working hours/day, schedule, number of breaks beyond regular breaks
3. Identification of work tasks	Text box for listing tasks
4. Expected productivity objective	Text box for listing the expected productivity level
5. Anticipated obstacles or difficulties	Text box for listing obstacles or difficulties the employee or employer anticipate
6. Interventions to compensate for the anticipated obstacles	Text box for listing interventions on an
	a) individual level
	b) organizational level
7. Employee’s level of confidence of fulfilling the work plan	Four-point rating scale
	1. not certain at all
	2. somewhat certain
	3. certain
	4. very certain
8. Signatures	Boxes for date and employer’s and employee’s signatures
At follow-up
9. Attainment of productivity objective	Three-point rating scale for attainment of objective
	1. Did not attain objective
	2. Partly attained objective
	3. Fully attained objective
10. Level of discomfort	Eleven-point rating scale, 0–10
0 = no discomfort, 10 = worst imaginable discomfort

## 4 Discussion

Our aim was to develop a Swedish version, GRTWswe, for implementation and integration into the Swedish labor market’s RTW process. This involved translating, culturally adapting, and assessing the appropriateness and utility of a Canadian tool called TS-GRTW.

The key findings indicate the need for revisions in the Swedish version of the instruction guide and planning worksheet. These changes are required due to contextual and cultural disparities between Canada and Sweden, especially in organizational structures, reference frameworks, and policy documents used in various vocational rehabilitation units. To begin, one cultural factor influencing the RTW process are the varying roles and responsibilities of different actors in different countries. In the original planning worksheet, a physician provided restrictions/recommendations for the employee. In Sweden, the SSIA has the main responsibility for the RTW process, assessing the needs of RTW interventions and coordinating the process with other stakeholders. The SSIA investigates the need for work-oriented measures and coordinates efforts from health and medical care services (i.e., OTs and physicians), the employer, SPES, social services, and other actors to enable the person to return to work or to look for work. This may, for example, refer to participating in a reconciliation meeting called by the SSIA. Consequently, in the planning worksheet, the ‘responsible physician’ should be changed to be based on a “plan for RTW from SSIA”.

All participants expressed a sense of unfamiliarity with the current 4-point scale used to rate level of discomfort and would prefer a scale with which they were already familiar. Scales commonly employed in pain rehabilitation within Sweden typically encompass 11-point structures, such as visual analogue scales, highlighting a prominent aspect for positive progress, and these are used both in research, in quality registers and in clinics. To address this and increase familiarity with the scale in the Swedish version, we will revise the 4-point scale of section 10 in the original version and adopt an 11-point scale instead, ranging from ‘no discomfort’ to ‘worst imaginable discomfort’. We will be able to evaluate the change to an 11-point scale and the usability from a user perspective in a forthcoming study that is set to commence in workplaces soon.

An advantage of the TS-GRTW is the evidence-based foundation and the core concept MM [10]. MM is a multidimensional concept and has been operationalized into six dimensions, namely, work context; employers’ requirements and expectations; means and tools; worker’s personal parameters; work activity; and impact of the work situation [16, 17]. Although some participants in the present study were not familiar with the concept MM, this is regulated for the Swedish labor market in The Swedish Work Environment Authority [26], where the definition of MM is described as: “the employer must ensure that employees have opportunities to influence the organization and implementation of their own work, so that they have sufficient movement variation and recovery”. The GRTWswe focuses on MM regulations in the Swedish RTW process. To enhance our understanding of MM in Sweden, we need to refine the Instruction guide for MM. It is essential to distinguish between this guide and the specialized initial margin of manoeuvre assessment guide for OTs [15, 16].

While the primary intention of TS-GRTW is for the tool to be employed by employers in conjunction with employees, the participants in this study recognized the potential to engage employees through various intermediaries, including OTs at specialty clinics and rehabilitation coordinators in primary care. These individuals can be seen as central coordinators who, during return-to-work meetings, establish connections with both employers and Occupational Health Care (OHC) representatives. Through this approach, they facilitate the implementation of the tool in various workplaces.

An interesting finding in this study was the agreement among participants to expand the tool’s target groups for employees regardless of diagnosis, and the clinical relevance of the tool to increase the transferability and usability of the tool. GRTWswe’s clinical relevance extends beyond chronic pain, accommodating return-to-work, unemployment, and new job situations, as well as aiding employers in supporting employees’ return, regardless of their diagnosis. This would also be in line with what Bouffard et al. [10] proposed regarding further developments of the TS-GRTW. According to the authors, there is a new trend toward developing tools in promoting a successful RTW that can be used for all types of diagnoses [10].

This study demonstrated that GRTWswe can help bridge the current gap in providing support to employers and employees during the RTW process. The tool encourages ongoing collaboration between supervisors and individual employees, facilitating communication, cooperation, and the creation of a shared plan through mutual decision-making [9]. In this study, the tool’s ability to enhance communication was highlighted during group consultations, similar to findings from the original Canadian version [10]. It was underscored that GRTWswe encourages communication among the parties involved. Enhanced communication and collaborative decision-making can also assist employers [7] in fulfilling their responsibilities within Swedish workplace context. In addition to fostering communication, the planning worksheet within GRTW promotes collaboration between the employer and employee [10]. TS-GRTW prioritizes continuous follow-up as a core objective, effectively addressing challenges that may arise during the RTW process through joint efforts. The importance of ongoing follow-up is underscored as one of the eight actionable strategies for facilitating employee progress in the RTW process [27], alongside crucial elements like assistance and planning. TS-GRTW conveniently integrates these essential steps. The study highlights how employees play a crucial role in managing their situation, emphasizing their responsibility, active involvement, and self-confidence. Motivation and their belief in their ability to handle pain also impacts collaboration [28]. This collaboration, in turn, can enhance self-efficacy, leading to improved outcomes [29, 30]. TS-GRTW can be seen as a self-management intervention, incorporating elements like goal setting, action planning, collaborative decision-making, and proactive follow-up [31]. Consequently, TS-GRTW strengthens self-efficacy, resulting in improved work-related outcomes. A supportive work environment plays a vital role in empowering individuals to engage in self-management strategies [31]. In summary, stakeholder agreement on a RTW goal and acceptance of an intervention plan that aligns task demands with worker capacity is crucial for successful RTW [32].

### 4.1 Strengths and Limitations

The study design was used to gather information from the participants, using both a questionnaire for the individual consultation and an interview for the group consultation. The questionnaire provided an overview and served as a foundation for subsequent discussions. Group interviews allowed participants to engage in more advanced and in-depth discussions, which is a notable strength of the study [33].

The OTs participating in this study possess specialized expertise in the intersection of chronic pain and work rehabilitation. It’s important to note that while these OTs may not have had an extensive familiarity with the MM concept itself, their evaluation primarily centered around the cultural adaptation of MM and its applicability. In the context of evaluating cultural adaptation, participants’ familiarity, and experience with the specific target group, as well as their experiences with vocational rehabilitation, are deemed adequate.

However, it is worth highlighting that the examination of the content validity of GRTWswe wasn’t feasible within the scope of this study because of the participants’ limited knowledge of MM. Despite this limitation; a notable strength of the study stems from the involvement of the original authors of the TS-GRTW. Their inclusion ensured that any modifications made to the tool-maintained alignment with the foundational principles of MM. This involvement added a layer of rigor and authenticity to the adaptation process, ultimately contributing to the study’s robustness.

A potential risk of group interviews is participants responding in a socially desirable manner, introducing bias. To counteract this, participants filled out surveys individually with their own comments before group interviews. They received interview topics in advance to prepare. Asking participants to write answers before discussions, as suggested by Sim [33], ensured comprehensive opinions were included. Additionally, having the same moderator (GML) for both groups provided consistent structure and content, strengthening the study.

A notable limitation of the study is the relatively small number of participants, which restricts the generalizability of the findings to a broader range of OTs. Nevertheless, it is important to acknowledge that the study was still able to capture a diverse array of viewpoints, given the participants’ distinct professional backgrounds. Furthermore, the inclusion of participants from five different geographical regions in the southern part of Sweden adds an element of geographical diversity to the sample. This geographical dispersion potentially enhances the study’s credibility by reflecting insights from various local contexts and practices within Sweden’s southern regions.

## 5 Conclusion

In summary, the study’s results highlighted strong agreement between the questionnaire responses and the outcomes of group consultations regarding the usability of the preliminary translated GRTWswe tool, demonstrating its suitability for application within a Swedish setting after some cultural adaptations. The tool offers distinct advantages by aiding both employers and employees. This is achieved through fostering communication, promoting collaborative efforts, and facilitating a structured process, all of which have promising prospects for establishing enduring and favorable work conditions. Moreover, there is potential to broaden the tool’s application to encompass other demographic groups encountering work-related issues, Additionally, diverse occupational groups beyond employers stand to gain considerable benefits from its application.

## Ethical approval

The study was approved by the Swedish Ethical Review Board (Dnr 2021-06678-01).

## Informed consent

The participating occupational therapists provided written informed consent, and the data were securely stored in a confidential database.

## Conflict of interest

The authors declare no conflict of interest.
